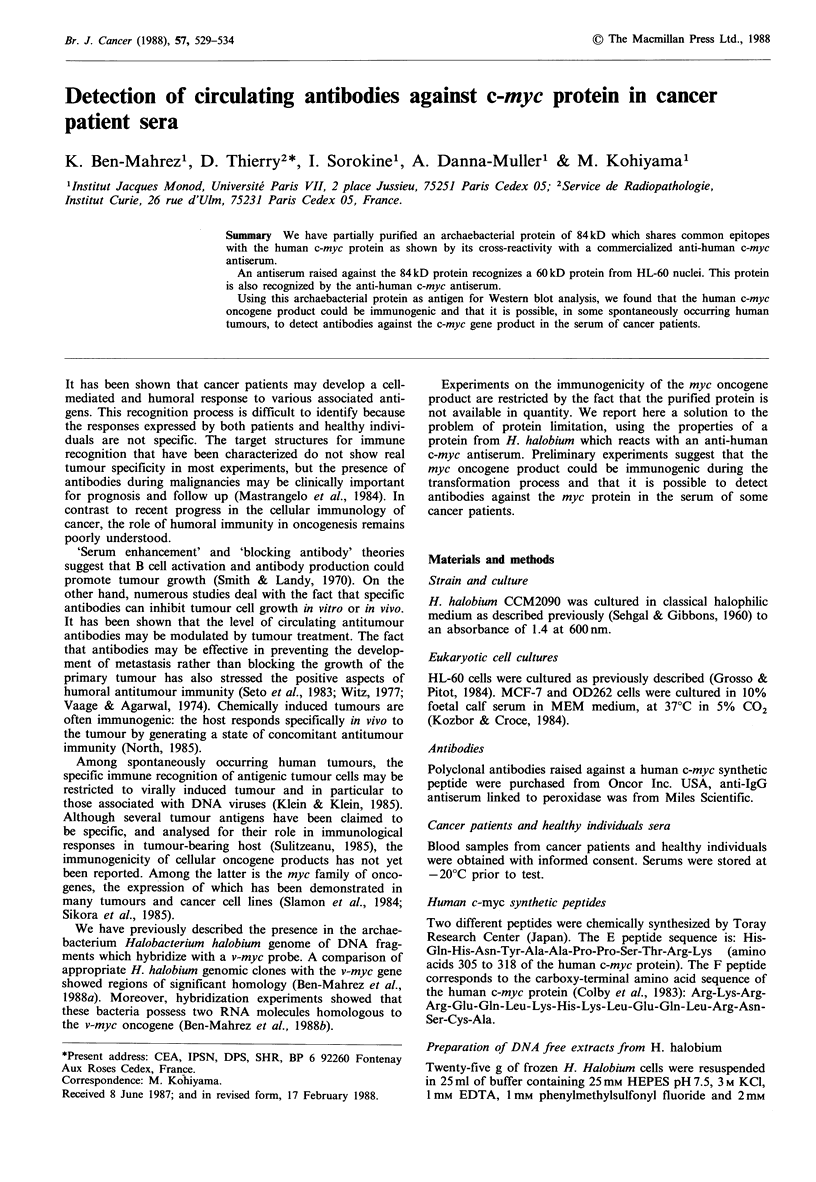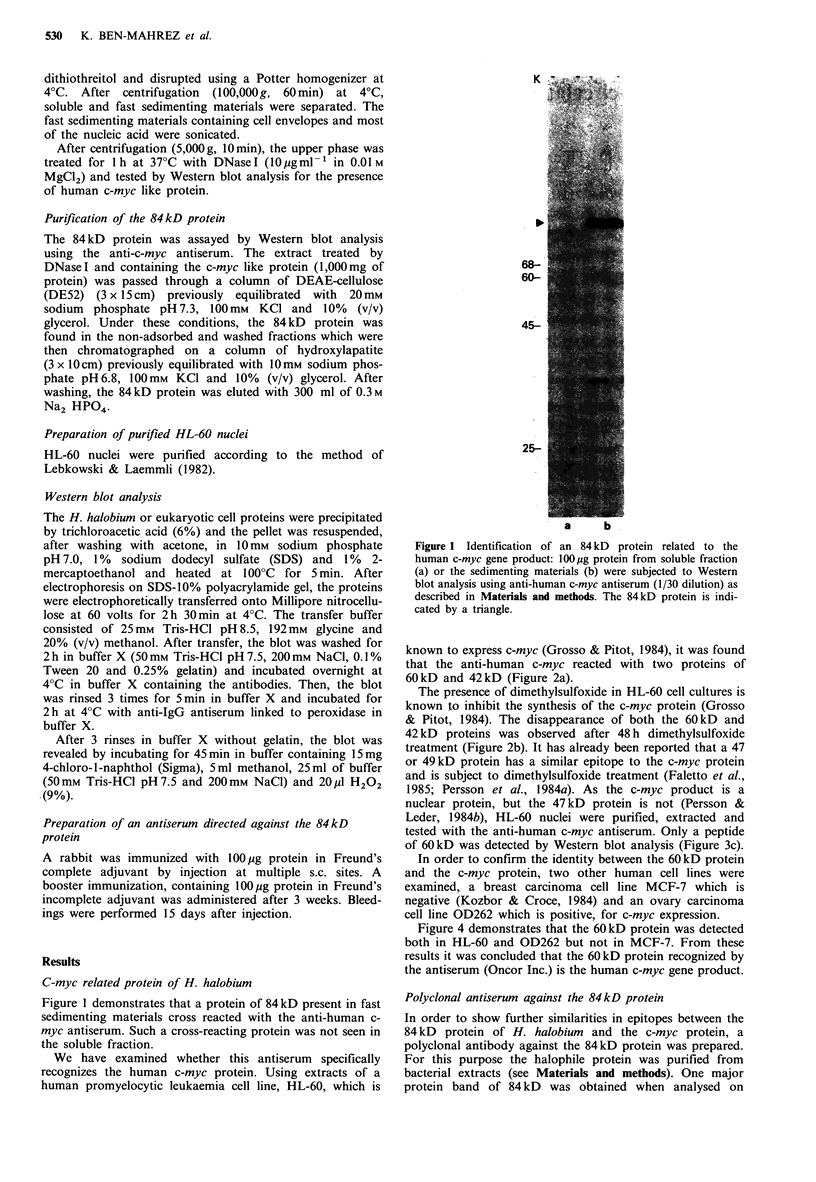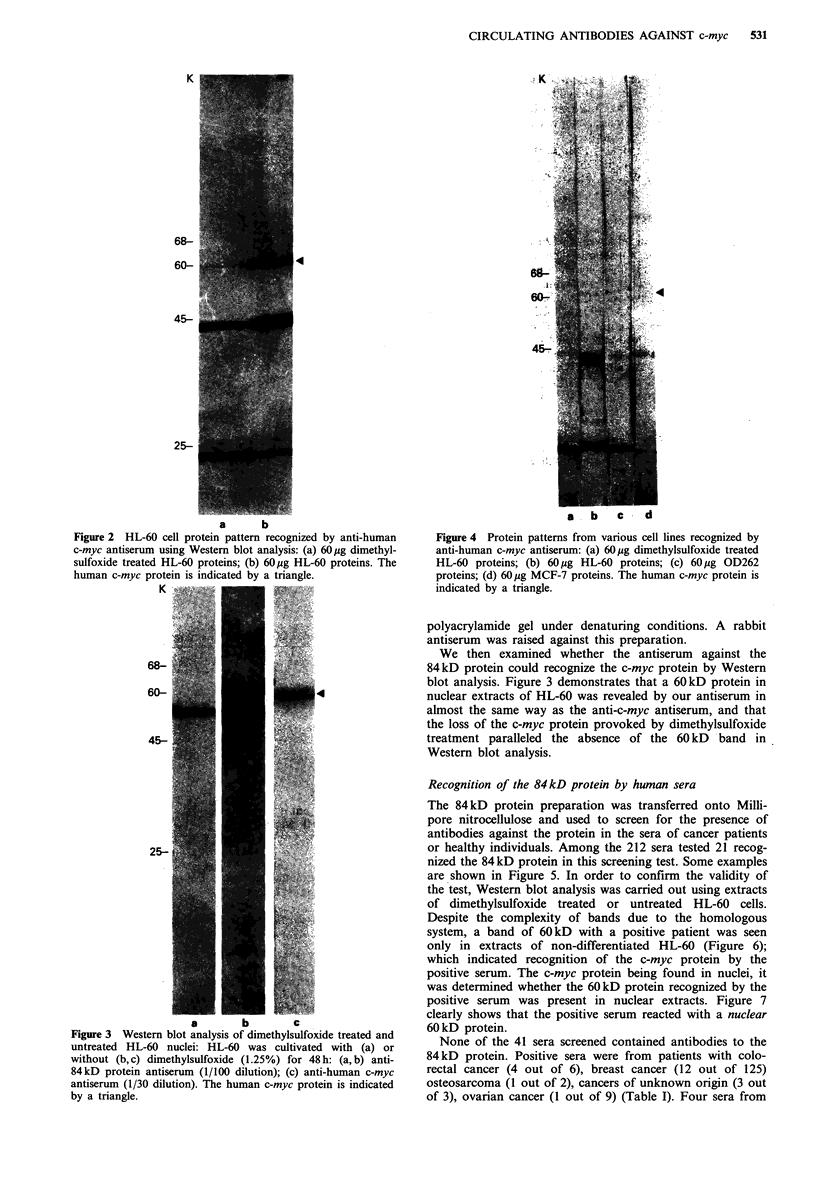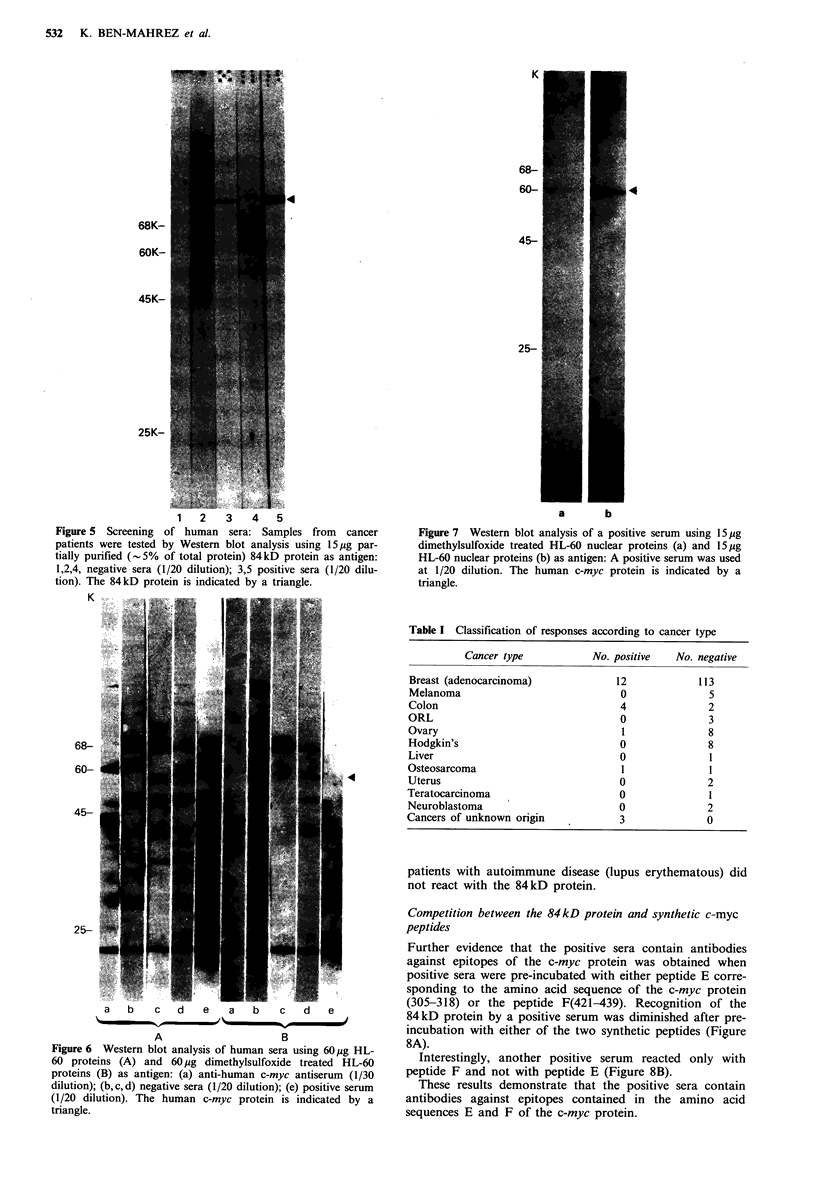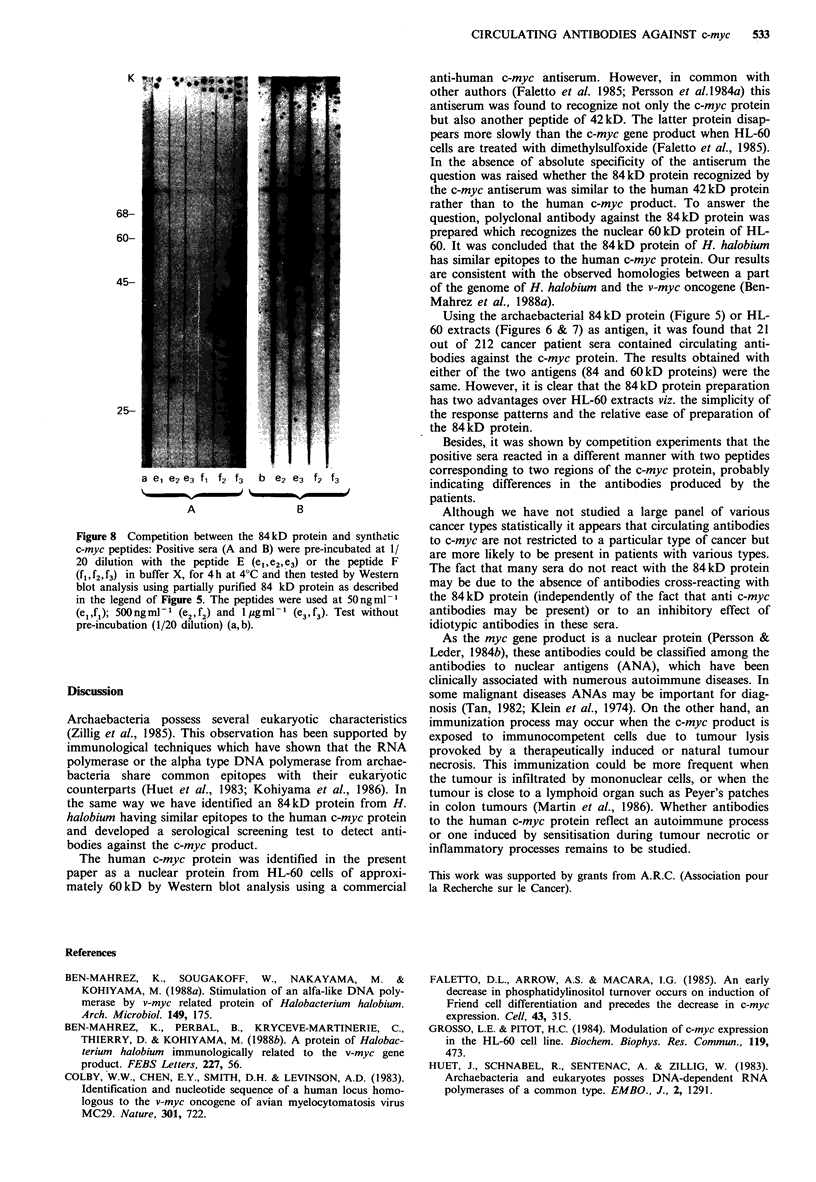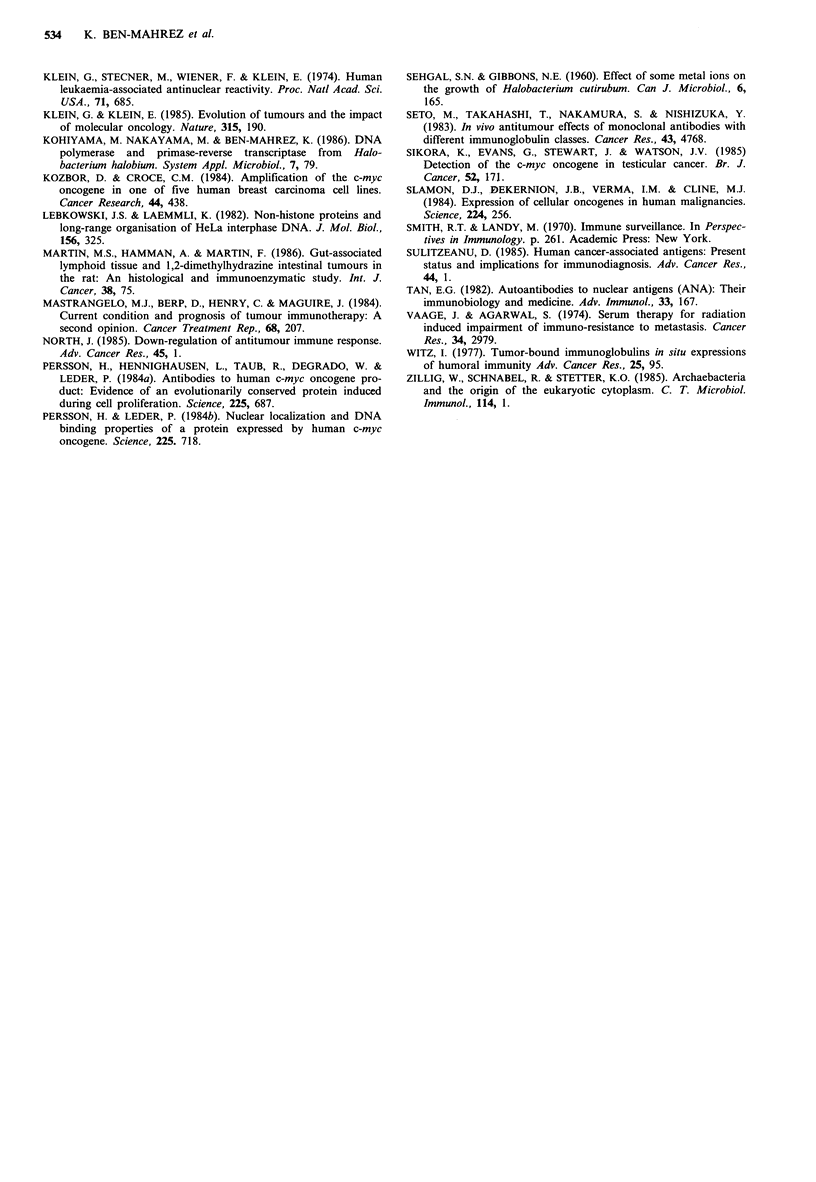# Detection of circulating antibodies against c-myc protein in cancer patient sera.

**DOI:** 10.1038/bjc.1988.123

**Published:** 1988-06

**Authors:** K. Ben-Mahrez, D. Thierry, I. Sorokine, A. Danna-Muller, M. Kohiyama

**Affiliations:** Institut Jacques Monod, UniversitÃ© Paris VII, France.

## Abstract

**Images:**


Br. J. Cancer (1988), 57, 529–534 © The Macmillan Press Ltd., 1988

### Detection of circulating antibodies against c-*myc* protein in cancer patient sera

K. Ben-Mahrez[1], D. Thierry[2]*, I. Sorokine[1], A. Danna-Muller[1] & M. Kohiyama[1]

[1]*Institut Jacques Monod, Université Paris VII, 2 place Jussieu, 75251 Paris Cedex 05;* [2]*Service de Radiopathologie, Institut Curie, 26 rue d'Ulm, 75231 Paris Cedex 05, France.*

**Summary** We have partially purified an archaebacterial protein of 84 kD which shares common epitopes with the human c-*myc* protein as shown by its cross-reactivity with a commercialized anti-human c-*myc* antiserum.

An antiserum raised against the 84 kD protein recognizes a 60 kD protein from HL-60 nuclei. This protein is also recognized by the anti-human c-*myc* antiserum.

Using this archaebacterial protein as antigen for Western blot analysis, we found that the human c-*myc* oncogene product could be immunogenic and that it is possible, in some spontaneously occurring human tumours, to detect antibodies against the c-*myc* gene product in the serum of cancer patients.

It has been shown that cancer patients may develop a cell-mediated and humoral response to various associated antigens. This recognition process is difficult to identify because the responses expressed by both patients and healthy individuals are not specific. The target structures for immune recognition that have been characterized do not show real tumour specificity in most experiments, but the presence of antibodies during malignancies may be clinically important for prognosis and follow up (Mastrangelo *et al.*, 1984). In contrast to recent progress in the cellular immunology of cancer, the role of humoral immunity in oncogenesis remains poorly understood.

'Serum enhancement' and 'blocking antibody' theories suggest that B cell activation and antibody production could promote tumour growth (Smith & Landy, 1970). On the other hand, numerous studies deal with the fact that specific antibodies can inhibit tumour cell growth *in vitro* or *in vivo*. It has been shown that the level of circulating antitumour antibodies may be modulated by tumour treatment. The fact that antibodies may be effective in preventing the development of metastasis rather than blocking the growth of the primary tumour has also stressed the positive aspects of humoral antitumour immunity (Seto *et al.*, 1983; Witz, 1977; Vaage & Agarwal, 1974). Chemically induced tumours are often immunogenic: the host responds specifically *in vivo* to the tumour by generating a state of concomitant antitumour immunity (North, 1985).

Among spontaneously occurring human tumours, the specific immune recognition of antigenic tumour cells may be restricted to virally induced tumour and in particular to those associated with DNA viruses (Klein & Klein, 1985). Although several tumour antigens have been claimed to be specific, and analysed for their role in immunological responses in tumour-bearing host (Sulitzeanu, 1985), the immunogenicity of cellular oncogene products has not yet been reported. Among the latter is the *myc* family of oncogenes, the expression of which has been demonstrated in many tumours and cancer cell lines (Slamon *et al.*, 1984; Sikora *et al.*, 1985).

We have previously described the presence in the archaebacterium *Halobacterium halobium* genome of DNA fragments which hybridize with a *v-myc* probe. A comparison of appropriate *H. halobium* genomic clones with the *v-myc* gene showed regions of significant homology (Ben-Mahrez *et al.*, 1988*a*). Moreover, hybridization experiments showed that these bacteria possess two RNA molecules homologous to the *v-myc* oncogene (Ben-Mahrez *et al.*, 1988*b*).

Experiments on the immunogenicity of the *myc* oncogene product are restricted by the fact that the purified protein is not available in quantity. We report here a solution to the problem of protein limitation, using the properties of a protein from *H. halobium* which reacts with an anti-human c-*myc* antiserum. Preliminary experiments suggest that the *myc* oncogene product could be immunogenic during the transformation process and that it is possible to detect antibodies against the *myc* protein in the serum of some cancer patients.

### Materials and methods

### Strain and culture

*H. halobium* CCM2090 was cultured in classical halophilic medium as described previously (Sehgal & Gibbons, 1960) to an absorbance of 1.4 at 600 nm.

### Eukaryotic cell cultures

HL-60 cells were cultured as previously described (Grosso & Pitot, 1984). MCF-7 and OD262 cells were cultured in 10% foetal calf serum in MEM medium, at 37°C in 5% $CO_2$ (Kozbor & Croce, 1984).

### Antibodies

Polyclonal antibodies raised against a human c-*myc* synthetic peptide were purchased from Oncor Inc. USA, anti-IgG antiserum linked to peroxidase was from Miles Scientific.

### Cancer patients and healthy individuals sera

Blood samples from cancer patients and healthy individuals were obtained with informed consent. Serums were stored at −20°C prior to test.

### Human c-myc synthetic peptides

Two different peptides were chemically synthesized by Toray Research Center (Japan). The E peptide sequence is: His-Gln-His-Asn-Tyr-Ala-Ala-Pro-Pro-Ser-Thr-Arg-Lys (amino acids 305 to 318 of the human c-*myc* protein). The F peptide corresponds to the carboxy-terminal amino acid sequence of the human c-*myc* protein (Colby *et al.*, 1983): Arg-Lys-Arg-Arg-Glu-Gln-Leu-Lys-His-Lys-Leu-Glu-Gln-Leu-Arg-Asn-Ser-Cys-Ala.

### Preparation of DNA free extracts from H. halobium

Twenty-five g of frozen *H. Halobium* cells were resuspended in 25 ml of buffer containing 25 mM HEPES pH 7.5, 3 M KCl, 1 mM EDTA, 1 mM phenylmethylsulfonyl fluoride and 2 mM

*Present address: CEA, IPSN, DPS, SHR, BP 6 92260 Fontenay Aux Roses Cedex, France.
Correspondence: M. Kohiyama.
Received 8 June 1987; and in revised form, 17 February 1988.

dithiothreitol and disrupted using a Potter homogenizer at 4°C. After centrifugation (100,000*g*, 60 min) at 4°C, soluble and fast sedimenting materials were separated. The fast sedimenting materials containing cell envelopes and most of the nucleic acid were sonicated.

After centrifugation (5,000 g, 10 min), the upper phase was treated for 1 h at 37°C with DNase I (10 $\mu g\,ml^{-1}$ in 0.01 M $MgCl_2$) and tested by Western blot analysis for the presence of human c-*myc* like protein.

*Purification of the 84 kD protein*

The 84 kD protein was assayed by Western blot analysis using the anti-c-*myc* antiserum. The extract treated by DNase I and containing the c-*myc* like protein (1,000 mg of protein) was passed through a column of DEAE-cellulose (DE52) (3 x 15 cm) previously equilibrated with 20 mM sodium phosphate pH 7.3, 100 mM KCl and 10% (v/v) glycerol. Under these conditions, the 84 kD protein was found in the non-adsorbed and washed fractions which were then chromatographed on a column of hydroxylapatite (3 x 10 cm) previously equilibrated with 10 mM sodium phosphate pH 6.8, 100 mM KCl and 10% (v/v) glycerol. After washing, the 84 kD protein was eluted with 300 ml of 0.3 M $Na_2\ HPO_4$.

*Preparation of purified HL-60 nuclei*

HL-60 nuclei were purified according to the method of Lebkowski & Laemmli (1982).

*Western blot analysis*

The *H. halobium* or eukaryotic cell proteins were precipitated by trichloroacetic acid (6%) and the pellet was resuspended, after washing with acetone, in 10 mM sodium phosphate pH 7.0, 1% sodium dodecyl sulfate (SDS) and 1% 2-mercaptoethanol and heated at 100°C for 5 min. After electrophoresis on SDS-10% polyacrylamide gel, the proteins were electrophoretically transferred onto Millipore nitrocellulose at 60 volts for 2 h 30 min at 4°C. The transfer buffer consisted of 25 mM Tris-HCl pH 8.5, 192 mM glycine and 20% (v/v) methanol. After transfer, the blot was washed for 2 h in buffer X (50 mM Tris-HCl pH 7.5, 200 mM NaCl, 0.1% Tween 20 and 0.25% gelatin) and incubated overnight at 4°C in buffer X containing the antibodies. Then, the blot was rinsed 3 times for 5 min in buffer X and incubated for 2 h at 4°C with anti-IgG antiserum linked to peroxidase in buffer X.

After 3 rinses in buffer X without gelatin, the blot was revealed by incubating for 45 min in buffer containing 15 mg 4-chloro-1-naphthol (Sigma), 5 ml methanol, 25 ml of buffer (50 mM Tris-HCl pH 7.5 and 200 mM NaCl) and 20 $\mu l$ $H_2O_2$ (9%).

*Preparation of an antiserum directed against the 84 kD protein*

A rabbit was immunized with 100 $\mu g$ protein in Freund's complete adjuvant by injection at multiple s.c. sites. A booster immunization, containing 100 $\mu g$ protein in Freund's incomplete adjuvant was administered after 3 weeks. Bleedings were performed 15 days after injection.

### Results

*C-myc related protein of H. halobium*

Figure 1 demonstrates that a protein of 84 kD present in fast sedimenting materials cross reacted with the anti-human c-*myc* antiserum. Such a cross-reacting protein was not seen in the soluble fraction.

We have examined whether this antiserum specifically recognizes the human c-*myc* protein. Using extracts of a human promyelocytic leukaemia cell line, HL-60, which is known to express c-*myc* (Grosso & Pitot, 1984), it was found that the anti-human c-*myc* reacted with two proteins of 60 kD and 42 kD (Figure 2a).

**Figure 1** Identification of an 84 kD protein related to the human c-*myc* gene product: 100 $\mu g$ protein from soluble fraction (a) or the sedimenting materials (b) were subjected to Western blot analysis using anti-human c-*myc* antiserum (1/30 dilution) as described in **Materials and methods**. The 84 kD protein is indicated by a triangle.

The presence of dimethylsulfoxide in HL-60 cell cultures is known to inhibit the synthesis of the c-*myc* protein (Grosso & Pitot, 1984). The disappearance of both the 60 kD and 42 kD proteins was observed after 48 h dimethylsulfoxide treatment (Figure 2b). It has already been reported that a 47 or 49 kD protein has a similar epitope to the c-*myc* protein and is subject to dimethylsulfoxide treatment (Faletto *et al.*, 1985; Persson *et al.*, 1984*a*). As the c-*myc* product is a nuclear protein, but the 47 kD protein is not (Persson & Leder, 1984*b*), HL-60 nuclei were purified, extracted and tested with the anti-human c-*myc* antiserum. Only a peptide of 60 kD was detected by Western blot analysis (Figure 3c).

In order to confirm the identity between the 60 kD protein and the c-*myc* protein, two other human cell lines were examined, a breast carcinoma cell line MCF-7 which is negative (Kozbor & Croce, 1984) and an ovary carcinoma cell line OD262 which is positive, for c-*myc* expression.

Figure 4 demonstrates that the 60 kD protein was detected both in HL-60 and OD262 but not in MCF-7. From these results it was concluded that the 60 kD protein recognized by the antiserum (Oncor Inc.) is the human c-*myc* gene product.

*Polyclonal antiserum against the 84 kD protein*

In order to show further similarities in epitopes between the 84 kD protein of *H. halobium* and the c-*myc* protein, a polyclonal antibody against the 84 kD protein was prepared. For this purpose the halophile protein was purified from bacterial extracts (see **Materials and methods**). One major protein band of 84 kD was obtained when analysed on

**Figure 2** HL-60 cell protein pattern recognized by anti-human c-*myc* antiserum using Western blot analysis: (a) 60 μg dimethylsulfoxide treated HL-60 proteins; (b) 60 μg HL-60 proteins. The human c-*myc* protein is indicated by a triangle.

**Figure 3** Western blot analysis of dimethylsulfoxide treated and untreated HL-60 nuclei: HL-60 was cultivated with (a) or without (b, c) dimethylsulfoxide (1.25%) for 48 h: (a, b) anti-84 kD protein antiserum (1/100 dilution); (c) anti-human c-*myc* antiserum (1/30 dilution). The human c-*myc* protein is indicated by a triangle.

**Figure 4** Protein patterns from various cell lines recognized by anti-human c-*myc* antiserum: (a) 60 μg dimethylsulfoxide treated HL-60 proteins; (b) 60 μg HL-60 proteins; (c) 60 μg OD262 proteins; (d) 60 μg MCF-7 proteins. The human c-*myc* protein is indicated by a triangle.

polyacrylamide gel under denaturing conditions. A rabbit antiserum was raised against this preparation.

We then examined whether the antiserum against the 84 kD protein could recognize the c-*myc* protein by Western blot analysis. Figure 3 demonstrates that a 60 kD protein in nuclear extracts of HL-60 was revealed by our antiserum in almost the same way as the anti-c-*myc* antiserum, and that the loss of the c-*myc* protein provoked by dimethylsulfoxide treatment paralleled the absence of the 60 kD band in Western blot analysis.

*Recognition of the 84 kD protein by human sera*

The 84 kD protein preparation was transferred onto Millipore nitrocellulose and used to screen for the presence of antibodies against the protein in the sera of cancer patients or healthy individuals. Among the 212 sera tested 21 recognized the 84 kD protein in this screening test. Some examples are shown in Figure 5. In order to confirm the validity of the test, Western blot analysis was carried out using extracts of dimethylsulfoxide treated or untreated HL-60 cells. Despite the complexity of bands due to the homologous system, a band of 60 kD with a positive patient was seen only in extracts of non-differentiated HL-60 (Figure 6); which indicated recognition of the c-*myc* protein by the positive serum. The c-*myc* protein being found in nuclei, it was determined whether the 60 kD protein recognized by the positive serum was present in nuclear extracts. Figure 7 clearly shows that the positive serum reacted with a *nuclear* 60 kD protein.

None of the 41 sera screened contained antibodies to the 84 kD protein. Positive sera were from patients with colorectal cancer (4 out of 6), breast cancer (12 out of 125) osteosarcoma (1 out of 2), cancers of unknown origin (3 out of 3), ovarian cancer (1 out of 9) (Table I). Four sera from

Figure 5 Screening of human sera: Samples from cancer patients were tested by Western blot analysis using 15 $\mu$g partially purified ($\sim$5% of total protein) 84 kD protein as antigen: 1,2,4, negative sera (1/20 dilution); 3,5 positive sera (1/20 dilution). The 84 kD protein is indicated by a triangle.

Figure 6 Western blot analysis of human sera using 60 $\mu$g HL-60 proteins (A) and 60 $\mu$g dimethylsulfoxide treated HL-60 proteins (B) as antigen: (a) anti-human c-*myc* antiserum (1/30 dilution); (b, c, d) negative sera (1/20 dilution); (e) positive serum (1/20 dilution). The human c-*myc* protein is indicated by a triangle.

Figure 7 Western blot analysis of a positive serum using 15 $\mu$g dimethylsulfoxide treated HL-60 nuclear proteins (a) and 15 $\mu$g HL-60 nuclear proteins (b) as antigen: A positive serum was used at 1/20 dilution. The human c-*myc* protein is indicated by a triangle.

**Table I** Classification of responses according to cancer type

| *Cancer type* | *No. positive* | *No. negative* |
|---|---|---|
| Breast (adenocarcinoma) | 12 | 113 |
| Melanoma | 0 | 5 |
| Colon | 4 | 2 |
| ORL | 0 | 3 |
| Ovary | 1 | 8 |
| Hodgkin's | 0 | 8 |
| Liver | 0 | 1 |
| Osteosarcoma | 1 | 1 |
| Uterus | 0 | 2 |
| Teratocarcinoma | 0 | 1 |
| Neuroblastoma | 0 | 2 |
| Cancers of unknown origin | 3 | 0 |

patients with autoimmune disease (lupus erythematous) did not react with the 84 kD protein.

### *Competition between the 84 kD protein and synthetic c-*myc *peptides*

Further evidence that the positive sera contain antibodies against epitopes of the c-*myc* protein was obtained when positive sera were pre-incubated with either peptide E corresponding to the amino acid sequence of the c-*myc* protein (305–318) or the peptide F(421–439). Recognition of the 84 kD protein by a positive serum was diminished after pre-incubation with either of the two synthetic peptides (Figure 8A).

Interestingly, another positive serum reacted only with peptide F and not with peptide E (Figure 8B).

These results demonstrate that the positive sera contain antibodies against epitopes contained in the amino acid sequences E and F of the c-*myc* protein.

**Figure 8** Competition between the 84 kD protein and synthetic c-*myc* peptides: Positive sera (A and B) were pre-incubated at 1/20 dilution with the peptide E ($e_1,e_2,e_3$) or the peptide F ($f_1,f_2,f_3$) in buffer X, for 4 h at 4°C and then tested by Western blot analysis using partially purified 84 kD protein as described in the legend of **Figure 5**. The peptides were used at 50 ng ml$^{-1}$ ($e_1$,$f_1$); 500 ng ml$^{-1}$ ($e_2$,$f_2$) and 1 $\mu$g ml$^{-1}$ ($e_3$,$f_3$). Test without pre-incubation (1/20 dilution) (a, b).

### Discussion

Archaebacteria possess several eukaryotic characteristics (Zillig *et al.*, 1985). This observation has been supported by immunological techniques which have shown that the RNA polymerase or the alpha type DNA polymerase from archaebacteria share common epitopes with their eukaryotic counterparts (Huet *et al.*, 1983; Kohiyama *et al.*, 1986). In the same way we have identified an 84 kD protein from *H. halobium* having similar epitopes to the human c-*myc* protein and developed a serological screening test to detect antibodies against the c-*myc* product.

The human c-*myc* protein was identified in the present paper as a nuclear protein from HL-60 cells of approximately 60 kD by Western blot analysis using a commercial anti-human c-*myc* antiserum. However, in common with other authors (Faletto *et al.* 1985; Persson *et al.*1984*a*) this antiserum was found to recognize not only the c-*myc* protein but also another peptide of 42 kD. The latter protein disappears more slowly than the c-*myc* gene product when HL-60 cells are treated with dimethylsulfoxide (Faletto *et al.*, 1985). In the absence of absolute specificity of the antiserum the question was raised whether the 84 kD protein recognized by the c-*myc* antiserum was similar to the human 42 kD protein rather than to the human c-*myc* product. To answer the question, polyclonal antibody against the 84 kD protein was prepared which recognizes the nuclear 60 kD protein of HL-60. It was concluded that the 84 kD protein of *H. halobium* has similar epitopes to the human c-*myc* protein. Our results are consistent with the observed homologies between a part of the genome of *H. halobium* and the v-*myc* oncogene (Ben-Mahrez *et al.*, 1988*a*).

Using the archaebacterial 84 kD protein (Figure 5) or HL-60 extracts (Figures 6 & 7) as antigen, it was found that 21 out of 212 cancer patient sera contained circulating antibodies against the c-*myc* protein. The results obtained with either of the two antigens (84 and 60 kD proteins) were the same. However, it is clear that the 84 kD protein preparation has two advantages over HL-60 extracts *viz.* the simplicity of the response patterns and the relative ease of preparation of the 84 kD protein.

Besides, it was shown by competition experiments that the positive sera reacted in a different manner with two peptides corresponding to two regions of the c-*myc* protein, probably indicating differences in the antibodies produced by the patients.

Although we have not studied a large panel of various cancer types statistically it appears that circulating antibodies to c-*myc* are not restricted to a particular type of cancer but are more likely to be present in patients with various types. The fact that many sera do not react with the 84 kD protein may be due to the absence of antibodies cross-reacting with the 84 kD protein (independently of the fact that anti c-*myc* antibodies may be present) or to an inhibitory effect of idiotypic antibodies in these sera.

As the *myc* gene product is a nuclear protein (Persson & Leder, 1984*b*), these antibodies could be classified among the antibodies to nuclear antigens (ANA), which have been clinically associated with numerous autoimmune diseases. In some malignant diseases ANAs may be important for diagnosis (Tan, 1982; Klein *et al.*, 1974). On the other hand, an immunization process may occur when the c-*myc* product is exposed to immunocompetent cells due to tumour lysis provoked by a therapeutically induced or natural tumour necrosis. This immunization could be more frequent when the tumour is infiltrated by mononuclear cells, or when the tumour is close to a lymphoid organ such as Peyer's patches in colon tumours (Martin *et al.*, 1986). Whether antibodies to the human c-*myc* protein reflect an autoimmune process or one induced by sensitisation during tumour necrotic or inflammatory processes remains to be studied.

This work was supported by grants from A.R.C. (Association pour la Recherche sur le Cancer).

### References

BEN-MAHREZ, K., SOUGAKOFF, W., NAKAYAMA, M. & KOHIYAMA, M. (1988*a*). Stimulation of an alfa-like DNA polymerase by *v-myc* related protein of *Halobacterium halobium*. *Arch. Microbiol.* **149,** 175.

BEN-MAHREZ, K., PERBAL, B., KRYCEVE-MARTINERIE, C., THIERRY, D. & KOHIYAMA, M. (1988*b*). A protein of *Halobacterium halobium* immunologically related to the v-*myc* gene product. *FEBS Letters,* **227,** 56.

COLBY, W.W., CHEN, E.Y., SMITH, D.H. & LEVINSON, A.D. (1983). Identification and nucleotide sequence of a human locus homologous to the *v-myc* oncogene of avian myelocytomatosis virus MC29. *Nature,* **301,** 722.

FALETTO, D.L., ARROW, A.S. & MACARA, I.G. (1985). An early decrease in phosphatidylinositol turnover occurs on induction of Friend cell differentiation and precedes the decrease in c-*myc* expression. *Cell,* **43,** 315.

GROSSO, L.E. & PITOT, H.C. (1984). Modulation of c-*myc* expression in the HL-60 cell line. *Biochem. Biophys. Res. Commun.,* **119,** 473.

HUET, J., SCHNABEL, R., SENTENAC, A. & ZILLIG, W. (1983). Archaebacteria and eukaryotes posses DNA-dependent RNA polymerases of a common type. *EMBO., J.,* **2,** 1291.

KLEIN, G., STECNER, M., WIENER, F. & KLEIN, E. (1974). Human leukaemia-associated antinuclear reactivity. *Proc. Natl Acad. Sci. USA.*, **71**, 685.

KLEIN, G. & KLEIN, E. (1985). Evolution of tumours and the impact of molecular oncology. *Nature*, **315**, 190.

KOHIYAMA, M. NAKAYAMA, M. & BEN-MAHREZ, K. (1986). DNA polymerase and primase-reverse transcriptase from *Halobacterium halobium*. *System Appl. Microbiol.*, **7**, 79.

KOZBOR, D. & CROCE, C.M. (1984). Amplification of the c-*myc* oncogene in one of five human breast carcinoma cell lines. *Cancer Research*, **44**, 438.

LEBKOWSKI, J.S. & LAEMMLI, K. (1982). Non-histone proteins and long-range organisation of HeLa interphase DNA. *J. Mol. Biol.*, **156**, 325.

MARTIN, M.S., HAMMAN, A. & MARTIN, F. (1986). Gut-associated lymphoid tissue and 1,2-dimethylhydrazine intestinal tumours in the rat: An histological and immunoenzymatic study. *Int. J. Cancer*, **38**, 75.

MASTRANGELO, M.J., BERP, D., HENRY, C. & MAGUIRE, J. (1984). Current condition and prognosis of tumour immunotherapy: A second opinion. *Cancer Treatment Rep.*, **68**, 207.

NORTH, J. (1985). Down-regulation of antitumour immune response. *Adv. Cancer Res.*, **45**, 1.

PERSSON, H., HENNIGHAUSEN, L., TAUB, R., DEGRADO, W. & LEDER, P. (1984*a*). Antibodies to human c-*myc* oncogene product: Evidence of an evolutionarily conserved protein induced during cell proliferation. *Science*, **225**, 687.

PERSSON, H. & LEDER, P. (1984*b*). Nuclear localization and DNA binding properties of a protein expressed by human c-*myc* oncogene. *Science*, **225**. 718.

SEHGAL, S.N. & GIBBONS, N.E. (1960). Effect of some metal ions on the growth of *Halobacterium cutirubum*. *Can J. Microbiol.*, **6**, 165.

SETO, M., TAKAHASHI, T., NAKAMURA, S. & NISHIZUKA, Y. (1983). *In vivo* antitumour effects of monoclonal antibodies with different immunoglobulin classes. *Cancer Res.*, **43**, 4768.

SIKORA, K., EVANS, G., STEWART, J. & WATSON, J.V. (1985) Detection of the c-*myc* oncogene in testicular cancer. *Br. J. Cancer*, **52**, 171.

SLAMON, D.J., DEKERNION, J.B., VERMA, I.M. & CLINE, M.J. (1984). Expression of cellular oncogenes in human malignancies. *Science*, **224**, 256.

SMITH, R.T. & LANDY, M. (1970). Immune surveillance. In *Perspectives in Immunology*. p. 261. Academic Press: New York.

SULITZEANU, D. (1985). Human cancer-associated antigens: Present status and implications for immunodiagnosis. *Adv. Cancer Res.*, **44**, 1.

TAN, E.G. (1982). Autoantibodies to nuclear antigens (ANA): Their immunobiology and medicine. *Adv. Immunol.*, **33**, 167.

VAAGE, J. & AGARWAL, S. (1974). Serum therapy for radiation induced impairment of immuno-resistance to metastasis. *Cancer Res.*, **34**, 2979.

WITZ, I. (1977). Tumor-bound immunoglobulins *in situ* expressions of humoral immunity *Adv. Cancer Res.*, **25**, 95.

ZILLIG, W., SCHNABEL, R. & STETTER, K.O. (1985). Archaebacteria and the origin of the eukaryotic cytoplasm. *C. T. Microbiol. Immunol.*, **114**, 1.